# Clinical experiences with canakinumab as a treatment for autoinflammatory disorders

**DOI:** 10.1186/1546-0096-13-S1-P202

**Published:** 2015-09-28

**Authors:** C Saperia, P McAuley, J Raffaghello, S Fazlul Haque, G Sussman

**Affiliations:** 1 Gordon Sussman Clinical Research Inc., Toronto, Ontario, Canada

## Rationale

Patients seeking clinical treatment for urticaria, while presenting with elevated inflammatory markers, were found to be suffering from auto-inflammatory disorders, such as Cryopyrin Associated Periodic Syndromes (CAPS), Familial Mediterranean Fever (FMF) and Schnitzler's syndrome. Patients were screened for, and/or began treatment with canakinumab.

## Methods

The 32 patients identified for investigation presented with cases of chronic urticaria along with some of the following symptoms: joint and bone pain, chills, conjunctivitis, periodic fever, fatigue, weight loss, and hearing loss. 11 patients (34%) presented with Muckle-Wells syndrome. Seven patients (22%) presented with Familial Cold Autoinflammatory Syndrome and a history of treatment with rilanocept. Three patients (9.5%) presented with FMF. Three patients (9.5%) presented with Schnitzler's syndrome. Eight patients (25%) presented with undiagnosed forms of CAPS. All patients showed elevated inflammatory markers, including Serum Amyloid A (SAA) and C-reactive protein (CRP). Nine patients with Muckle-Wells syndrome and a confirmed R260W mutation at the NLRP3 gene, and one patient with an undiagnosed form of CAPS began treatment with canakinumab. Patients received a 150 mg treatment via sub-cutaneous injection every eight weeks. Total injections to date for individual patients range from two to seven. The remaining 22 patients are currently being investigated for treatment with canakinumab.

## Results

All ten patients treated with canakinumab experienced remission within one week of their first injection. Remissions were maintained by subsequent injections. Seven patients with SAA levels >16,000 ng/mL (normal range 1000-5000 ng/mL) prior to treatment showed normal SAA levels within one week of their first injection.

## Conclusions

Canakinumab treatment for patients with Muckle-Wells syndrome was associated with complete spontaneous remissions within one week of commencing treatment, and sustained clinical improvements over time. Patients presenting with chronic urticaria should be carefully assessed for disguised auto-inflammatory disorders, through genetic testing and examination for elevated inflammatory markers.

